# A standardised workflow to manage the complexity of reirradiation and radiotherapy retreatments in clinical practice^☆^

**DOI:** 10.1016/j.tipsro.2025.100336

**Published:** 2025-09-02

**Authors:** Madalyne Day, Jonas Willmann, Panagiotis Balermpas, Riccardo Dal Bello, Anja Joye, Laura Motisi, Jens von der Grün, Crystal Sulaiman, Lotte Wilke, Nazanin Rahnama, Matthias Guckenberger, Stephanie Tanadini-Lang, Nicolaus Andratschke

**Affiliations:** Department of Radiation Oncology, University Hospital Zurich and University of Zurich, Zurich, Switzerland

**Keywords:** Reirradiation, Workflow, Retreatment

## Abstract

As cancer patients survive longer, the number of radiotherapy retreatments is steadily increasing [1], requiring laborious review of previous treatments, decisions regarding image registration, dose accumulation and assessment of such composite dose distributions. This work presents the development, and implementation of a standardised dedicated workflow for reirradiation (reRT) and radiotherapy retreatment based on the EORTC/ESTRO consensus on reRT.

## Highlights

• Development and implementation of a standardised dedicated workflow for reirradiation, and radiotherapy retreatment.
• Integrated a hierarchical decision-making process to balance PTV coverage and cumulative OAR constraints.
• Workflow applied to 692 reirradiation cases, with an average simulation-to-treatment time of 7 working days.

## Short introduction

Due to continuous advancements in cancer therapy, the number of long-term cancer survivors has been steadily increasing [2] and radiotherapy, alongside surgical intervention and systemic therapies, remains one of the key pillars of modern cancer treatment. Following an initial course of radiotherapy, some patients may develop locoregional recurrences, metastases, or new primary tumours, sometimes in a previously irradiated area. In select cases, a local ablative treatment in curative intent is possible. However, these secondary treatments are often more complex and challenging than primary interventions, as patients may have toxicity from previous treatments, increasing the risk of such interventions, as well as the actual prognosis of such patients being difficult to assess.

In this context, reRT is gaining importance in clinical practice and trials [3,4]. Continuous advancements in treatment planning and precise image-guided radiotherapy enable more targeted and safer treatments, even in areas previously exposed to high radiation doses. However, the implementation of reRT in routine clinical practice is subject to numerous challenges [5,6]. On the technical side, standardised protocols and quality assurance tools for image registration, dose accumulation and calculation of equivalent dose in 2 Gy fractions (EQD2Gy) are lacking. Clinically, evidence for safe cumulative doses to organs at risk (OAR) and the potential of normal tissue recovery from subclinical radiation damage is limited. Currently, commercially available treatment planning systems (TPS) do not fully integrate the entire reRT workflow [6,7].

To ensure that safe and effective reRT can be delivered in clinical practice, standardised workflows are crucial. However, with few exceptions [8], published reports on reRT workflows are scarce. This work presents the development and implementation of a standardised dedicated workflow for reRT and radiotherapy retreatment at the radiation oncology department of the University Hospital of Zurich based on the EORTC/ESTRO consensus on reRT.

## Materials and methods

A core team of radiation oncologists (n = 5), radiation therapist/dosimetrists (n = 2), with additional support from senior medical physics staff (n = 3) defined clear and detailed guidelines for reRT: An overall workflow with responsibilities for the individual workflow steps, image registration, EQD2Gy-based dose accumulation (including alpha/beta ratios), cumulative allowance for OAR doses (Table 1), steps for preparation and treatment planning, and final decision making were developed. The reRT OAR tolerances listed in Table 1 are based on in-house expert consensus of which are currently under evaluation in a prospective cohort to determine the safety of these dose limits. The workflow choice was informed by reviewing available literature, clinical experience within the team, and available technical tools.

**Table 1:** Departmental OAR tolerances used in the reirradiation workflow. All tolerance doses are defined in EQD2. Structures that end in + 1 mm require a planning risk volume of 1 mm to be added. ‘Open’ refers to an open-ended constraint, and ‘no constraint’ refers to OARs that have no defined constraints, both of which should be discussed on a case-by-case basis. Values are based on in-house expert consensus of which are currently under evaluation in a prospective cohort to determine the safety of these dose limits.

**Structure**	**Alpha/Beta**	**Parameter**	**Eq2Gy**	
			**Total Dose [Gy]**	
			**Objective**	**Variation**
**Thorax**				
Lungs	3	Dmean [Gy]	≤ 22.00	≤ 26.00
Lungs	3	D1500cc	≤ 22.00	≤ 26.00
Lung	3	V20 [Gy]	≤ 40 %	
Brachial Plexus	3	D 3.0 cc [Gy]	≤ 75.00	≤ 90.00
Brachial Plexus	3	D0.03 cc [Gy]	≤ 90.00	≤ 110.00
Bronchial Tree	3	D0.1 cc [Gy]	≤ 120.00	≤ 140.00
Bronchial Tree	3	D0.03 cc [Gy]	≤ 130.00	≤ 150.00
Bronchial Tree	3	D5cc [Gy]	≤ 90.00	≤ 95.00
Thoracic Wall	3	D30cc [Gy]	≤ 80.00	≤ 100.00
Thoracic Wall	3	D5cc [Gy]	≤ 120.00	≤ 140.00
Esophagus	3	D0.03 cc [Gy]	≤ 100.00	≤ 120.00
Esophagus	3	D0.1 cc [Gy]	≤ 90.00	≤ 110.00
Esophagus	3	D1.00 cc [Gy]	≤ 80.00	≤ 90.00
Esophagus	3	D5.00 cc [Gy]	≤ 70.00	≤ 90.00
Heart	3	D15cc [Gy]	≤ 60.00	≤ 80.00
Heart	3	D0.1 cc [Gy]	≤ 85.00	≤ 130.00
Spinal Canal	2	D0.1 cc [Gy]		
Great Vessels	3	D0.03 cc [Gy]	≤ 120.00	open
**Abdomen**				
Kidney	3	D200cc [Gy]	≤ 15.00	≤ 20.00
Kidney	3	D75%	≤ 12.00	≤ 15.00
Stomach	3	D0.1 cc [Gy]	≤ 60.00	≤ 80.00
Stomach	3	D1cc [Gy]	≤ 54.00	≤ 70.00
Duodenum	3	D0.1 cc [Gy]	≤ 60.00	≤ 80.00
Duodenum	3	D1cc [Gy]	≤ 54.00	≤ 70.00
Bowel	3	D0.1 cc [Gy]	≤ 60.00	≤ 80.00
Bowel	3	D0.5 cc [Gy]	≤ 54.00	≤ 70.00
Liver-GTV	3	D700cc [Gy]	≤ 18.00	≤ 25.00
Liver-GTV	3	Dmean [Gy]	≤ 25.00	≤ 30.00
Spinal Nerve	2	D0.1 cc [Gy]	≤ 70.00	≤ 90.00
Cauda Equina	3	D0.1 cc [Gy]	≤ 67.00	≤ 85.00
Sacral Plexus	2	D0.1 cc [Gy]	≤ 67.00	≤ 85.00
**Pelvis**				
Bladder	3	D0.5 cc [Gy]	≤ 105.00	≤ 120.00
Bladder	3	D2cc [Gy]	≤ 95.00	≤ 105.00
Rectum	3	D0.5 cc [Gy]	≤ 105.00	≤ 110.00
Rectum	3	D2cc [Gy]	≤ 95.00	≤ 105.00
Spinal Nerve	2	D0.03 cc [Gy]	≤ 90.00	≤ 110.00
Spinal Nerve	2	D1cc [Gy]	≤ 75.00	≤ 90.00
Ureter	3	no constraint		
Urethra	3	no constraint		
Femoral Head	3	no constraint		
Penile Bulb	3	no constraint		
**Head and Neck**				
Brainstem	2	D0.1 cc [Gy]	≤ 68.00	≤ 80.00
Chiasm	2	D0.03 cc [Gy]	≤ 68.00	≤ 80.00
Cochlea ipsilateral	3	Dmean [Gy]	≤ 45.00	≤ 60.00
Cochlea contralateral	3	Dmean [Gy]	≤ 45.00	≤ 60.00
Optic Nerve	2	D0.03 cc [Gy]	≤ 68.00	≤ 80.00
Trachea	3	D0.03 cc [Gy]	≤ 120.00	≤ 140.00
Trachea	3	D0.1 cc [Gy]	≤ 110.00	≤ 120.00
Brain	3	D0.1 cc [Gy]	≤ 110.00	≤ 120.00
Carotid Artery + 1 mm	3	D0.03 cc [Gy]	≤ 100.00	≤ 135.00
Mandible	3	Dmean [Gy]	≤ 40.00	≤ 60.00
Mandible	3	D0.1 cc [Gy]	≤ 70.00	≤ 130.00
**Brain**				
Brainstem	2	D0.1 cc [Gy]	≤ 80.00	≤ 100.00
Chiasm	2	D0.03 cc [Gy]	≤ 68.00	≤ 80.00
Optic Nerve	2	D0.03 cc [Gy]	≤ 68.00	≤ 80.00
Retina	3	D0.03 cc [Gy]	≤ 70.00	≤ 80.00
Lens	3	no constraint		
Brain − GTV	3	D0.1 cc [Gy]	≤ 100.00	≤ 120.00

All work required for this workflow was performed within ARIA (Varian Medical Systems, A Siemens Healthineers Company) as the record and verify system, and Eclipse (v16.1) TPS (Varian Medical Systems, A Siemens Healthineers Company). Within ARIA a tool is available to organise tasks, and dedicated workflows can be created. A specific reRT workflow was developed for all treatment courses categorised as type 1 and type 2 reRT. ReRT type 1 refers to situations where there is an overlap of a previous radiotherapy course with the new course of treatment, whereas type 2 does not have an overlap of the previous course and new course; however, there is a concern for toxicity. All other situations of a further course of radiotherapy are considered ‘repeat organ irradiation’ or ‘repeat irradiation’, neither of which cause concern for toxicity. Due to the critical nature of type 1 and 2 reRT, these cases were selected for our dedicated workflow. The reirradiation types were assigned by the treating oncologist according to the ESTRO/EORTC consensus on reirradiation [9]. General disease characteristics, and the time from simulation to treatment start was captured. This work was approved by the cantonal ethics committee (BASEC ID 2018–01794).

## Results

During the period of March 2023 − October 2024, 5475 treatment courses were completed, of which 692 were Type 1 and 2 reRT (13 %). Of these Type 1 and 2 reirradiation courses, bone (27.0 %) and central nervous system (23.3 %) were the most common anatomical regions to be treated with the distribution displayed in Fig. 3. The average time from patient simulation to the first treatment for this cohort was 7 working days.

### Collection of previous treatment information

During initial consultation with the patient the treating radiation oncologist will confirm the reRT status of the patient. For patients treated externally, the treating radiation oncologist will initiate the administration and physics team via email to request the RTdata, which will be imported and registered with any relevant documentation, while the responsible physicist will import relevant data into the treatment planning system. This requires written consent from the patient and cooperation with other clinics as there are no frameworks nationally or internationally to access previous treatment information. Patients that were previously treated within the institution may have all previous treatment information available, however, some data may have been archived digitally, or only in paper form. Where possible, previous radiotherapy plans, corresponding planning CT scans, and radiotherapy structure sets in DICOM format, as well as relevant documentation will be collected and imported.

For either situation, if DICOM data is not available it may be requested to recreate the treatment plan based on paper records of treatment fields or dose distributions, or assume a homogeneous dose over the entire treatment field as recommended by the ESTRO/EORTC best practice recommendation [9]. The radiation oncologist will record the patient’s reRT type and all previous treatments in the local electronic health record of the patient. A comprehensive naming protocol is implemented within the clinic, which allows clear identification using increasing course numbers to indicate further irradiations, as well as numbering of tumour volumes, which are increasing for each new volume, but are maintained if a patient were to return for treatment of a recurrence within a previously treated volume.

This information is also utilised by radiation therapists performing the simulation to guide positioning for the reRT. Where possible, the patient will be positioned in a similar way to the initial treatment to facilitate easier image registration with previous images in cases where this does not hinder the safety or reproducibility of the new treatment.

### Feasibility evaluation

An intended prescription dose for the new treatment will be provided by the treating radiation oncologist based on available clinical guidelines or clinical judgment. A treatment planner assesses the feasibility of reRT by accumulating the dose delivered in relevant previous treatments and evaluates if standardised OAR dose constraints specific for reirradiation can be met *(*Table 1*)*. If possible, this is performed using 3D dose mapping on the new planning image within the TPS. All previous treatment plans need to be converted to EQD2Gy, where an alpha/beta of 2 Gy is used for the central nervous system, including spinal cord, and an alpha/beta of 3 Gy for all other normal tissues. As image registration in the reRT setting can be particularly challenging—due to anatomical changes such as tumour regression, postoperative alterations, or the (dis)appearance of tissues within the treatment region—a rigorous and standardised registration process was implemented to ensure the accuracy of accumulated dose estimation. Only rigid image registration (RIR) is currently used, with alignment focused on stable bony landmarks such as the vertebral bodies, sacrum, or pelvic bones, and adjusted to ensure the alignment is especially accurate in the areas where high radiation doses were delivered or are planned, rather than trying to perfectly align the entire anatomy. This is because accurate alignment in these critical regions is most important for evaluating the combined effect of previous and current radiation treatments. In-house quality control projects of which the data is unpublished, as well as the difficulty of performing patient specific quality assurance for deformable image registration led to the decision to perform RIR only. This approach is in line with findings of an international survey where most other clinics also perform RIR for dose accumulation in the reRT setting [3]. Each registration is performed by the treatment planner and is visually reviewed together with a supervising medical physicist. The trustworthiness of the RIR, and consequently the accumulated dose, is recorded in the electronic patient record, an example of the table used is shown in Supplementary Material 1. If the RIR is deemed untrustworthy for a given OAR, a manual estimate based on summed maximum or near-maximum doses will be performed for a conservative estimate [9].

Following this process, the residual dose allowance per OAR can be determined. Higher cumulative dose constraints compared to the initial course of radiotherapy may be acceptable for certain organs. This could be due to assumed recovery (e.g. for the spinal cord), or secondary to higher acceptance of toxicity risks in the reRT situation.

### Treatment planning, dose accumulation, and plan approval

Treatment planning is performed according to an in-house dedicated reRT planning protocol using the isodose lines of the accumulated previous doses as help structures in the optimisation. The specific isodose lines that are used are different for each plan and are based on what the previous dose was, and the OAR tolerance that needs to be achieved. At this stage, the reRT workflow follows a structured, hierarchical decision-making approach to balance target volume coverage with OAR protection depicted in Fig. 2.

At the **first level**, the feasibility of achieving standard OAR dose constraints without compromising the planning target volume (PTV) is assessed. If this is possible, treatment planning proceeds according to the standard radiotherapy optimisation workflow (i.e. like de novo radiotherapy). If not, the **second level** is initiated, where reRT-specific OAR constraints from the departmental OAR tolerance guidelines were used.

At this **second level**, the treatment planner evaluates whether *Objective* OAR tolerances can be met, which achieves full PTV coverage without compromise. If these stricter thresholds are not achievable, the *Variation* tolerances are considered. If the *Variation* tolerances can be met, PTV coverage remains uncompromised. However, if even the *Variation* thresholds cannot be achieved, PTV compromise is then permitted within predefined limits, provided that minimum target coverage criteria were met. Specifically, for conventional external beam radiotherapy (EBRT), a minimum V95% > 80 % for GTV/CTV/ITV is required, and for stereotactic body radiotherapy/stereotactic radiosurgery (SBRT/SRS), a minimum GTV coverage of V100% > 80 % is mandated.

If *Variation* tolerances still cannot be met, even with allowed PTV compromise, the workflow escalates to the **third level**, which requires a modification of the treatment prescription. In this final step, PTV compromise was accepted until the reRT-specific OAR tolerances are fulfilled, in discussion with the treating radiation oncologist. For the spinal cord, specifically, tolerance is calculated based on the initial dose and time since prior radiotherapy, based on the formula by Nieder et al. [10]. This formula assumes partial time-dependent recovery of the spinal cord. No recovery is considered for other OARs.

Final doses per OAR are recorded in the patient file (either based on 3D dose mapping, or conservative manual point dose summation in the case of unreliable image registration; the type of dose accumulation is also recorded).

### Peer review and quality assurance

**Fig. 1:**
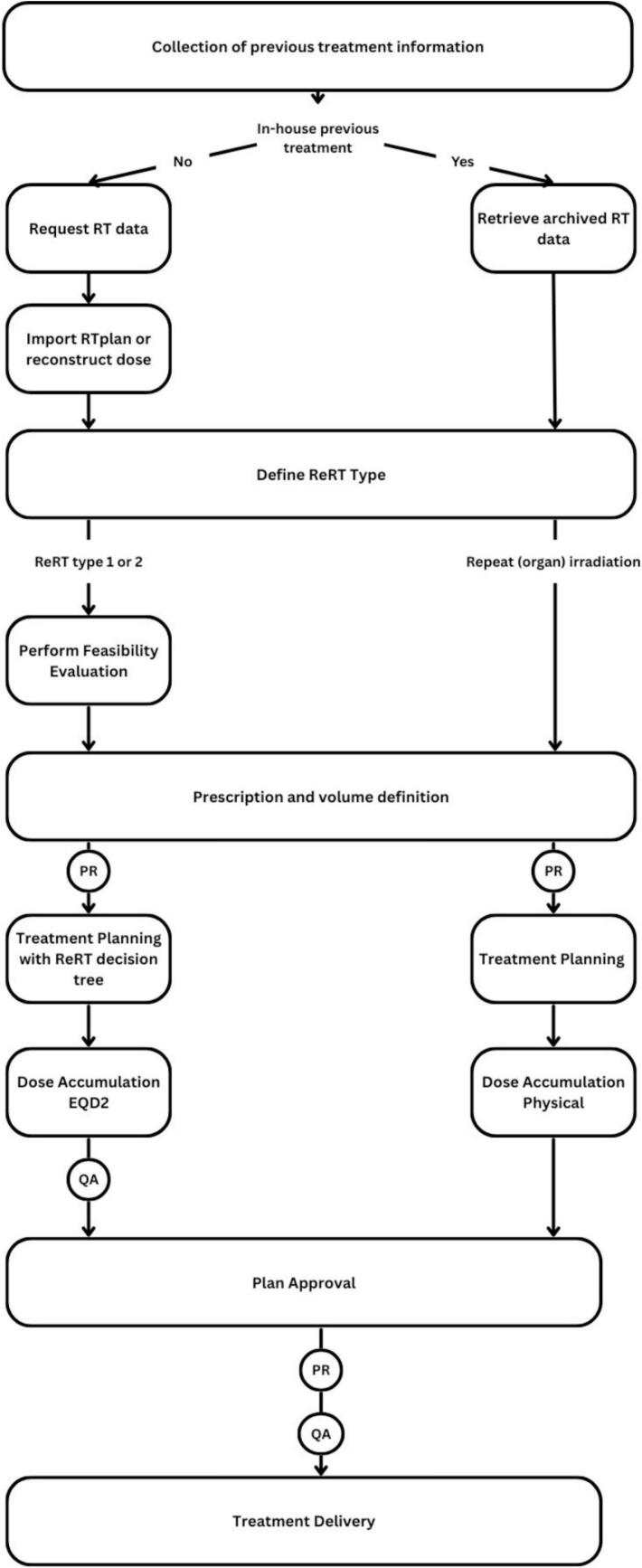
Dedicated workflow for reirradiation and radiotherapy retreatment. RT = Radiation Therapy; ReRT = Reirradiation; PR = Peer Review by a panel of radiation oncologists, a medical physicist, and a radiation therapist EQD2 = Equieffective dose in 2 Gy; QA = Quality assurance performed according to in-house protocol and national guidelines.

A standardised practise for quality control of treatment plans involves a peer review of the intended prescription and volumes, as well as the final accepted treatment plan. These are presented at two time points (Fig. 1) to an in-house panel of radiation oncologists, a medical physicist and a radiation therapist to ensure consensus within the team on all treatments. Quality assurance (QA) of the dose accumulation including the image fusion, manual calculation, and EQD2Gy calculations are performed by a medical physicist. The final patient specific QA is performed according to standard clinic protocols which are not specific to the reRT workflow.

## Discussion

Our institution has developed and implemented a dedicated reRT workflow that offers a structured, clinically pragmatic approach to the complex challenges of cumulative OAR dose constraints and target volume coverage.

We systematically categorise reRT cases based on the EORTC/ESTRO consensus on reRT, which allows us to provide a rational basis for workflow decision-making and facilitates communication among the multidisciplinary teams.

**Fig. 2:**
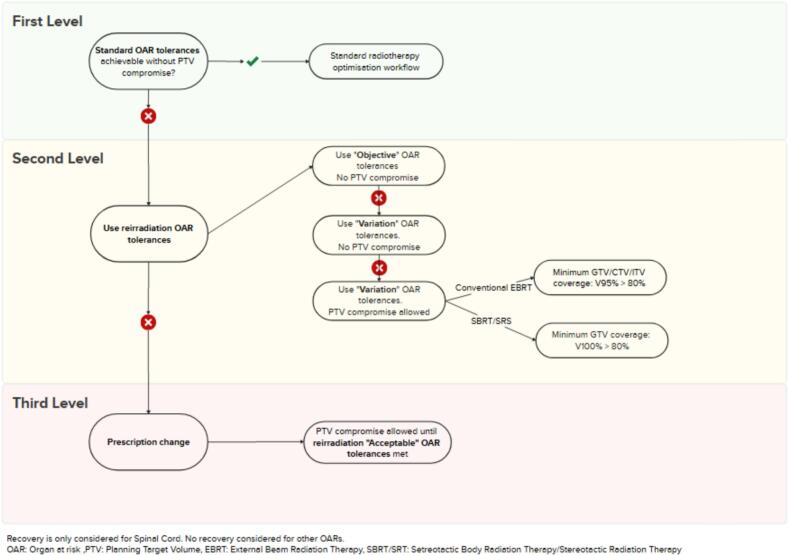
Reirradiation decision tree guides decision making for treatment planning of Type 1 and 2 reirradiation.

The implementation of a structured decision tree (Fig. 2) introduces a much-needed standardisation into the inherently complex reirradiation scenarios. By providing a clear and hierarchical framework for decision-making, the workflow avoids arbitrary or inconsistent clinical judgements, ensuring that compromises on target volume coverage are made systematically and transparently, rather than ad hoc.

**Fig. 3:**
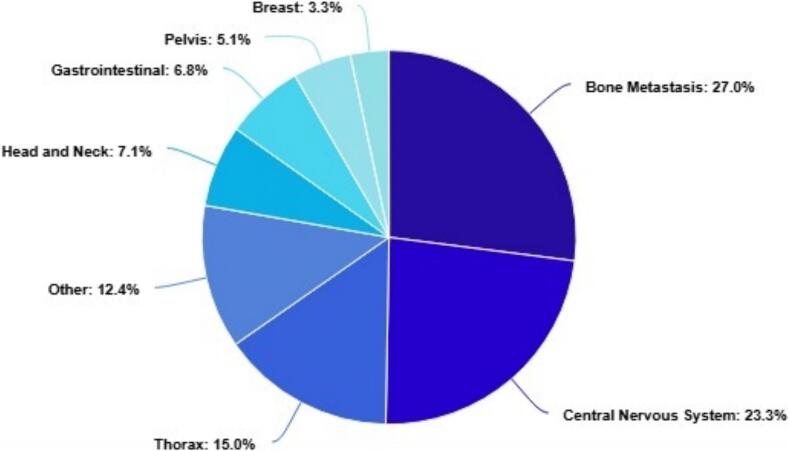
Reirradiation Type 1 and 2 patients treated between March 2023 − October 2024 categorised by treatment site. The category ‘other’ refers to the following indications skin, connective tissue, urinary system, male reproductive organs, gynaecological (excluding breast), unspecified lymph nodes, lymphomas, and leukaemia.

While the workflow has been successfully integrated at our institution, broader adoption will require addressing several critical challenges. Firstly, accurate assessment of cumulative doses requires robust image registration and dose summation workflows. As emphasised by Hardcastle et al. [7], establishing a standardised methodology for image registration (rigid vs. deformable) and dose accumulation is critical for reproducibility across institutions.

On the other hand, our cumulative OAR dose allowances, while systematically developed, require further clinical validation. Ongoing efforts, including in-house data collection initiatives and participation in the ReCare project (https://project.eortc.org/e2-radiate/cohorts/), aim to generate outcome data that will refine these thresholds.

Lastly, incorporating base-dose planning techniques enables optimisation of the reRT plan based on previously delivered doses. In particular, work by Murray et al. [11] provides a valuable foundation for base-dose–aware planning strategies, which we aim to further integrate into our treatment planning to enhance plan quality and safety.

In conclusion, our experience supports the feasibility and clinical value of implementing a structured, multidisciplinary reRT workflow, providing a foundation for timely, and reproducible retreatment in the modern radiation oncology setting.

## CRediT authorship contribution statement

**Madalyne Day:** Conceptualization, Writing – original draft, Writing – review & editing. **Jonas Willmann:** Writing – original draft, Writing – review & editing. **Panagiotis Balermpas:** Conceptualization, Writing – review & editing. **Riccardo Dal Bello:** Software, Writing – review & editing. **Anja Joye:** Data curation. **Laura Motisi:** Conceptualization, Writing – review & editing. **Jens von der Grün:** Conceptualization, Writing – review & editing. **Crystal Sulaiman:** Conceptualization, Writing – review & editing. **Lotte Wilke:** Writing – review & editing. **Nazanin Rahnama:** Writing – original draft, Writing – review & editing. **Matthias Guckenberger:** Writing – review & editing. **Stephanie Tanadini-Lang:** Conceptualization, Writing – review & editing. **Nicolaus Andratschke:** Conceptualization, Writing – original draft, Writing – review & editing.

## Declaration of competing interest

The authors declare the following financial interests/personal relationships which may be considered as potential competing interests: The department of Radiation Oncology of University Hospital of Zurich has research and teaching agreements with Siemens Healthineers and had research agreements with Viewray Inc.

Jonas Willmann is supported by the Swiss National Science Foundation (P500PM_203194) and reports speaker honoraria from RadFormation.

Stephanie Tanadini-Lang received speaker honoraria from Siemens Healthineers.

Madalyne Day received research funding from Viewray Inc.

All remaining authors have declared no conflicts of interest.
