# Supplementary material for: A standardised workflow to manage the complexity of reirradiation and radiotherapy retreatments in clinical practice
Source: Tech Innov Patient Support Radiat Oncol. 2025 Sep 2;35:100336. doi: 10.1016/j.tipsro.2025.100336 (PMC12441716; doi:10.1016/j.tipsro.2025.100336)
Supplement: Supplementary Data 1 [file mmc1.docx]

# Supplementary Material


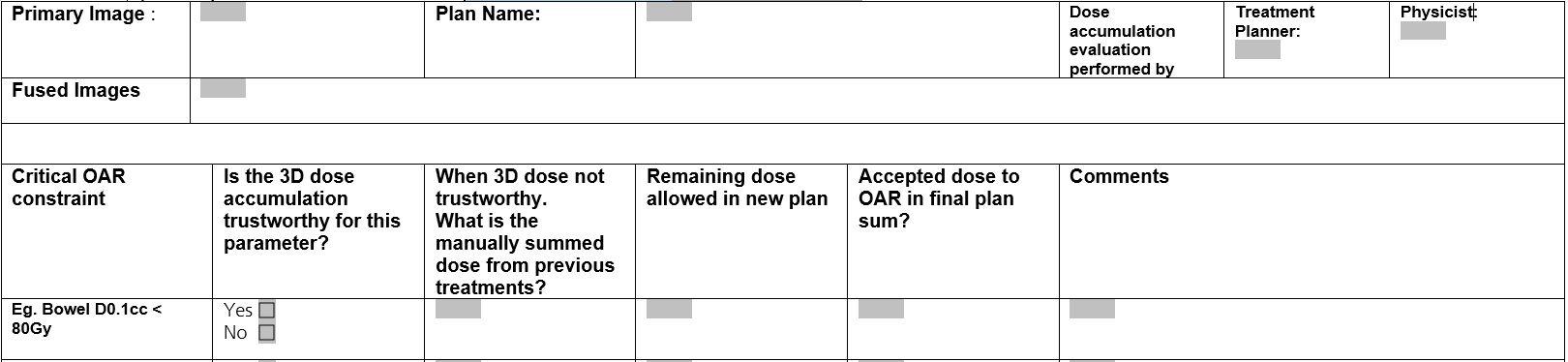


Supplementary Material 1. Table used for recording image fusion information in patient file.

This is a retrospective case study. Patient consent has been waived by Ethic committee.
